# STUDIES ON ADJUSTMENT TO RETIREMENT: HOW OLDER EUROPEANS NAVIGATE THE RETIREMENT TRANSITION

**DOI:** 10.1093/geroni/igz038.076

**Published:** 2019-11-08

**Authors:** van Solinge, Olga Grunwald

**Affiliations:** 1 Netherlands Interdisciplinary Demographic Institute, The Hague, Netherlands; 2 NIDI, Den Haag, South Holland, Netherlands

## Abstract

Retirement is a significant life transition in late adult life that often brings about great changes in individuals’ patterns of everyday activity, social networks as well as one’s economic resources, requiring adjustment for both the retiree and other members of the household. Retirement is a process that starts with a preparatory stage, followed by the actual act of retirement and a post-retirement stage where retirees have to get used to the changing aspects of life that result from the work-retirement transition, and seek to achieve psychological comfort with their retirement life. This symposium brings together empirical research on the various stages of the retirement process, from different national backgrounds. The guiding question is how work and the loss of work affect well-being. Hence, the symposium will give insights into the circumstances under which retirement risks well-being and psychological comfort of older adults. Anna Wanka discusses under which conditions retirement feels right for German retirees, and how this feeling shifts and changes throughout the retirement process. Sarah Dury follows with the post-retirement stage by demonstrating a qualitative perspective of recently retired Belgians about their adjustment, role and activities they exert during post-retirement. Isabelle Hansson examines the role of personality for retirement adjustment in a Swedish sample of older adults. Hanna van Solinge explores the impact of agency in the work-retirement transition on adjustment to a longer working life /retirement and life satisfaction in a Dutch panel study.

